# Chronic high-fat diet decreases global histone H4 acetylation and increases HDAC8 expression in mouse testes

**DOI:** 10.1016/j.bbrep.2026.102642

**Published:** 2026-05-20

**Authors:** Shu Aizawa, Hikari Ohno, Yutaka Yamamuro

**Affiliations:** Division of Physiology, Department of Zoological Sciences, College of Bioresource Sciences, Nihon University, Japan

**Keywords:** High-fat diet, Obesity, Histone acetylation, Histone deacetylase, Testis

## Abstract

Obesity is a major public health problem and a risk factor for metabolic disorders, including type 2 diabetes and cardiovascular disease. Additionally, accumulating evidence suggests that obesity impairs male reproductive capacity, potentially leading to infertility. However, molecular insights into the effects of obesity on the male reproductive system remain elusive. In this study, we examined the effects of diet-induced obesity on epigenetic marks—specifically histone acetylation—and the expression of epigenetic regulatory enzymes in mouse testes. C57BL/6 N male mice were randomly divided into two groups: a control group fed a control diet (10% kcal from fat) and a diet-induced obesity group fed a high-fat diet (HFD; 45% kcal from fat) for 8 weeks. Then, the acetylated histone H3 (K9 and K14) and histone H4 (K5, K8, K12, and K16) levels, as well as the expression of the histone deacetylase (HDAC) family, were investigated in the testes. We found that chronic HFD exposure decreased the acetylation levels of histone H4, but not histone H3, in the entire testis. We also observed the increased expression of HDAC8 at both the mRNA and protein levels in the testes of HFD-fed mice. HFD exposure did not affect the expression of other class I HDACs (HDAC1, HDAC2, and HDAC3) in the testes. These findings suggest that HFD-induced obesity disrupts epigenetic features in the testes, specifically through the modulation of HDAC8 and histone H4 acetylation, providing novel insight into obesity-induced male reproductive dysfunction.

## Highlights

• HFD decreased the levels of acetylated histone H4, but not histone H3, in the testes.
• HFD increased the expression of HDAC8 at the mRNA and protein levels in the testes.
• HFD-induced obesity affects the epigenetic information in the testes.

## 1 Introduction

The persistent increase in the number of overweight and obese individuals has been recognized as a global public health crisis [1,2]. Obesity significantly increases the risk of various non-communicable diseases, including type 2 diabetes, cardiovascular disease, and certain types of cancer [3]. Furthermore, numerous epidemiological and animal studies have demonstrated that metabolic disturbances, such as obesity, profoundly affect reproductive health. Specifically, obesity in males is strongly associated with reduced testosterone levels, impaired spermatogenesis, and compromised sperm quality, establishing it as a major risk factor for male infertility [4]. However, the molecular mechanisms by which environmental factors, such as nutritional conditions, impair testicular tissue homeostasis remain largely unknown.

Throughout the differentiation of spermatogonia into mature spermatozoa in the testis, epigenetic regulation, which involves DNA methylation and posttranslational histone modification, is indispensable for maintaining genome stability and appropriate gene expression patterns in germ cells [5,6]. Furthermore, it is believed that epigenetic regulation is indispensable for the proper histone-to-protamine exchange during spermatogenesis [7]. Previous studies have demonstrated that environmental factors, including nutritional disturbances, can alter epigenetic signatures in the testis. Fullston et al. [8] showed decreased global DNA methylation in the testes of diet-induced obese mice, particularly in elongated spermatids. More recently, Sukur et al. [9] histologically confirmed that diet-induced obesity decreases global DNA methylation, which is accompanied by altered expression of DNA methyltransferase 1 (DNMT1) and DNMT3a in the testes. Diet-induced obesity in mice also affects the methylation and acetylation of histone H3 and the acetylation of histone H4 in the entire testis [10]. Other researchers have reported that feeding mice a high-fat diet (HFD) decreases the ubiquitination of histone H2A and the acetylation of histone H4 in the entire testis [11]. These findings suggest that diet-induced obesity causes epigenetic changes in the testes and leads to reproductive dysfunction. However, there is a lack of research on the regulatory molecules of diet-induced epigenetic changes, especially the enzymes that support posttranslational histone modifications in the testes.

To gain further insight into the molecules regulating diet-induced epigenetic changes in the testes, we examined the impact of an HFD on global histone acetylation and the expression of histone-modifying enzymes in the testes of mice. We found that HFD-induced obesity decreases the acetylation levels of histone H4 but not histone H3 in the testes. Furthermore, we discovered that HFD exposure specifically increases the gene and protein expression of histone deacetylase 8 (HDAC8) in the testes of mice.

## 2 Materials and methods

### 2.1 Animals

All animal experiments were approved by the Nihon University Animal Care and Use Committees and performed under the Guidelines for Animal Experiments, College of Bioresource Sciences, Nihon University (approval number: AP20BRS064-2). Four-week-old C57BL/6 N male mice were purchased from Japan SLC, Inc. (Shizuoka, Japan) and housed in an animal experimental facility with a 12 h-light/12 h dark cycle (lights on at 09:00) at 23 ± 1 °C. Mice were randomly assigned to receive either a control diet (CD; 10% kcal from fat, 3.85 kcal/g, D12450H, Research Diet, Inc., New Brunswick, NJ, USA) or an HFD (45% kcal from fat, 4.73 kcal/g, D12451, Research Diet, Inc.) beginning at 5 weeks old and continuing for 8 weeks, as previously described [12]. At 13 weeks old, mice were sacrificed using CO_2_, and the epididymal white adipose tissue (eWAT), subcutaneous white adipose tissue (sWAT), and testes were harvested for examination.

### 2.2 Histology

Tissues were fixed with 10% Formalin Neutral Buffer Solution (FUJIFILM Wako Pure Chemical Corp., Osaka, Japan) by immersion for 24 h at 4 °C and embedded in paraffin wax. Tissue sections were cut (7 μm thick) and stained with hematoxylin and eosin (H&E) using Mayer's Hematoxylin Solution and 1% Eosin Y Solution (FUJIFILM Wako Pure Chemical Corp.). The sections were observed using a light microscope (BZ-X800, Keyence Corporation, Osaka, Japan).

### 2.3 Total RNA and protein extraction from testes

Testicular tissues were homogenized in TRIzol Reagent (Thermo Fisher Scientific, Waltham, MA, USA) using a homogenizer (Polytron PT 10-35 GT, Kinematica, Inc., Bohemia, NY, USA), and total RNAs were extracted according to the manufacturer's instructions. Total proteins were isolated from the resulting phenol-ethanol phase according to the method reported by Kopec et al. [13]. Briefly, an excess amount of isopropanol was added to the phenol-ethanol solution, and proteins were precipitated by centrifugation. The protein pellets were washed twice with 95% ethanol and air dried for 10 min at room temperature. The pellets were resuspended in a buffer containing 10 mM Tris-HCl (pH 8.0), 140 mM NaCl, 1 mM EDTA (pH 8.0), and 5% SDS, and incubated for 2 h at 50 °C. The protein concentrations of each sample were quantified using a DC Protein Assay Kit (Bio-Rad Laboratories, Hercules, CA, USA), employing bovine serum albumin (BSA) as a standard.

### 2.4 Western blot analysis

Protein samples were separated by electrophoresis on SDS-polyacrylamide gels and transferred to Immobilon-P membranes (Merk Millipore, Burlington, MA, USA). After transfer, membranes were blocked with 3% BSA in phosphate-buffered saline (PBS) or Block ACE, ELISA Western Blotting Blocking Agent (KAC Co., Ltd., Kyoto, Japan). Then, the membranes were immunostained with Anti-acetyl-Histone H3 antibody, which detects acetyl K9 and K14, Anti-acetyl-Histone H4 antibody, which detects acetyl K5, K8, K12, and K16, Anti-HDAC1 antibody, Anti-HDAC2 antibody, Anti-HDAC3 antibody, Anti-HDAC8 antibody, or Anti-β-actin antibody as internal control. Detailed information about the antibodies used in this study, as well as the dilution ratio, is listed in Supplementary Table S1 (Table S1). After washing with PBS containing 0.1% Tween-20 (PBS-T), the membranes were incubated with HRP-conjugated secondary antibodies in PBS-T for an additional hour. Detection was performed using a LuminoGraph I system (ATTO Corp., Tokyo, Japan) and Luminata Forte Western HRP substrates (Merk Millipore). Band intensities were measured using ImageJ software (version 1.46r). Full-length blots of each protein are presented in Supplementary Fig. S1 (Fig. S1).

### 2.5 Real-time quantitative PCR analysis

Total RNAs obtained from testes were reverse transcribed using an iScript cDNA Synthesis Kit (Bio-Rad Laboratories). Real-time PCR was performed using a CFX Connect Real-time PCR Detection System (Bio-Rad Laboratories) and PowerUp SYBR Green Master Mix (Thermo Fisher Scientific). The primer sequences are listed in Supplementary Table S2 (Table S2). The PCR reaction involved 2 min of denaturation at 95 °C, followed by 40 cycles at 95 °C for 15 s, and then 60 °C for 1 min. After amplification, a melt curve analysis was performed to verify the authenticity of the amplified product based on its specific melting temperature. The 2^–ΔΔCt^ method was implemented to calculate the relative expression of genes by normalizing them to *18S ribosomal RNA* (*Rn18s*), with the control group serving as the calibrator.

### 2.6 Immunohistochemistry

For immunohistochemistry, mounted sections were subjected to antigen retrieval by heating in the Sodium Citrate Antigen Retrieval Buffer (Proteintech Group Inc., Rosemont, IL, USA) after deparaffinization and hydration. The endogenous peroxidase activity in the sections was blocked using the BLOXALL Endogenous Blocking Solution, Peroxidase and Alkaline Phosphatase (Vector Laboratories, Burlingame, CA, USA). Following several washes with PBS, sections were blocked by 3% BSA in PBS at room temperature for 30 min to prevent non-specific binding. Then, sections were incubated for 30 min at room temperature with the following primary antibodies: Anti-acetyl-Histone H4 antibody (Merck Millipore; 1:500) or Anti-HDAC8 antibody (Proteintech Group Inc.; 1:500). Several sections were incubated with Rabbit (DA1E) Monoclonal Antibody IgG Isotype Control (Cell Signaling Technology, Inc., Beverly, MA, USA; 1:500) to test primary antibody specificity. After several washes, sections were incubated with biotinylated goat anti-rabbit IgG (H + L) (Abcam, Cambridge, UK) at room temperature for 10 min, and then incubated with streptavidin peroxidase complex (Abcam). Finally, immunopositive signals were developed with 3,3′-diaminobenzidine (DAB) chromogen using the Rabbit specific HRP/DAB Detection Kit (Abcam) according to the manufacturer's instruction. All sections were dehydrated in a graded ethanol series, cleared in xylene, and covered with a Permount Mounting Medium (Fisher Scientific, Pittsburg, PA, USA). The sections were observed using a light microscope (BZ-X800, Keyence Corporation) as described previously.

### 2.7 Statistical analysis

Data are presented as the mean ± standard error of the mean (SEM). The significance of the difference between the two dietary groups was determined using Student's *t*-test and GraphPad Prism version 6.0 (GraphPad Software, Inc., San Diego, CA, USA), employing *P* < 0.05 as the criterion for significance in all cases.

## 3 Results

### 3.1 Effects of HFD on body weight gain, tissue weight, and testis histology

**Fig. 1:**
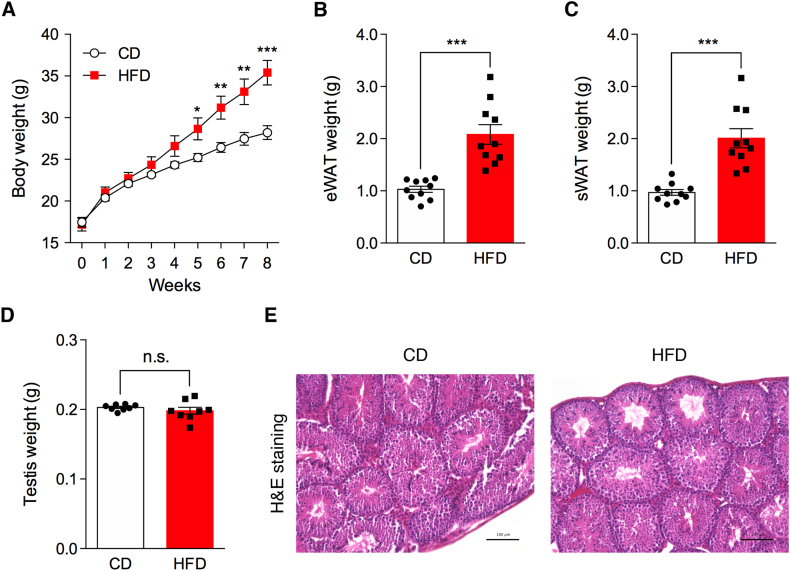
Establishment of an HFD-induced obese mouse model. (A) Body weight gain of male mice fed a CD or an HFD from 5 to 13 weeks of age. Data represent the mean ± SEM (n = 8). *P < 0.05, **P < 0.01, and ***P < 0.001 (Student's *t*-test). (B) eWAT, (C) sWAT, and (D) testes weights of male mice fed a CD or an HFD at 13 weeks of age. Data represent the mean ± SEM (n = 8). ***P < 0.001 (Student's *t*-test). n.s. indicates not significant. (E) Representative H&E staining of the testes from mice fed a CD (left) or an HFD (right). Scale bars indicate 100 μm.

To investigate the diet-induced epigenetic changes in the testis, 5-week-old male mice were fed either a CD or an HFD for 8 weeks. As previously reported [12], the HFD significantly increased the body weight in this model (Fig. 1A). The elevated body weight was significant at 5 weeks and remained significantly elevated for 3 weeks. After 8 weeks of HFD feeding, the eWAT and sWAT weights, as an index of adiposity, were remarkably increased compared with those after 8 weeks of CD feeding (Fig. 1B and C). In parallel with the adipose tissues, we measured the testicular weights of the CD- and HFD-fed mice, and found no significant difference between the two dietary groups (Fig. 1D). Histological images of the testes were also evaluated, and no morphological changes were observed in either group (Fig. 1E). These results demonstrate that HFD feeding in our experimental design induces obesity and causes mild metabolic stress in the testes.

### 3.2 HFD alters the acetylation levels of histone H4, but not histone H3, in the testes

**Fig. 2:**
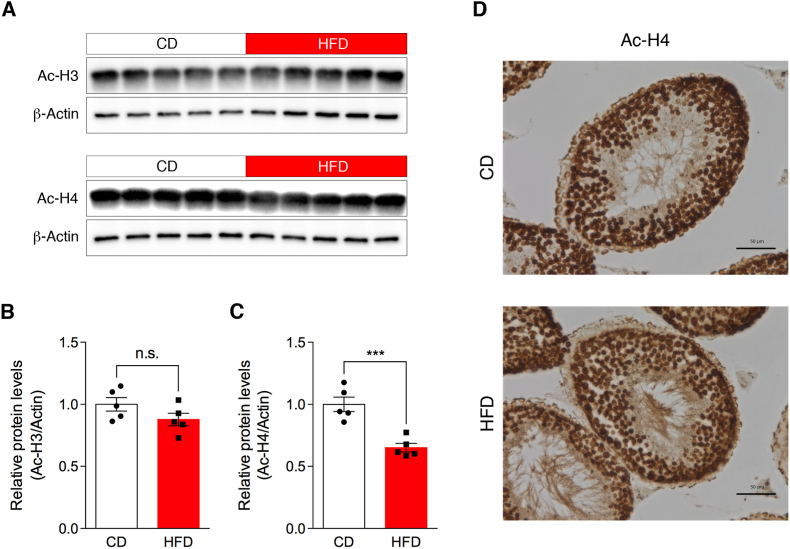
Effects of HFD on global histone H3 and H4 acetylation in the whole testis. (A) Western blot analysis of acetylated histone H3 and H4. Testicular tissue lysates from CD- and HFD-fed mice were subjected to western blotting with antibodies against Ac-H3 (K9 and K14) or Ac-H4 (K5, K8, K12, and K16). The equal loading of proteins was determined using an antibody against β-actin protein. Quantification of (B) Ac-H3 and (C) Ac-H4 levels in the testis. Band densities were measured using ImageJ software and normalized to β-actin protein. (D) Immunohistochemical analysis of acetylated histone H4 in the testes of mice fed either a CD (top) or an HFD (bottom). Scale bars indicate 50 μm. Data represent the mean ± SEM (n = 5). ***P < 0.001 (Student's *t*-test). n.s. indicates not significant.

To investigate the effects of HFD on testicular epigenetic marks, we focused on the histone acetylation levels. As shown in Fig. 2A, Western blot analysis revealed decreased levels of acetylated histone H4 (K5, K8, K12, and K16), but not acetylated histone H3 (K9 and K14), in the testes of HFD-fed mice. Quantification of the band density revealed a significant decrease in global histone H4 acetylation levels (Fig. 2B, *P* = 0.023), but no difference in global histone H3 acetylation levels (Fig. 2C). Immunohistochemical analysis showed that acetylated histone H4 was present in spermatogonia and throughout stages from primary spermatocytes to spermatids. Slightly lower levels of acetylated histone H4 were observed in the testis of mice fed an HFD (Fig. 2D). These results are consistent with previous reports that observed decreased histone H4 acetylation levels in whole testis lysates obtained from HFD-induced obese mice [10,11].

### 3.3 HFD increases expression of the HDAC8 gene and protein in the testes

**Fig. 3:**
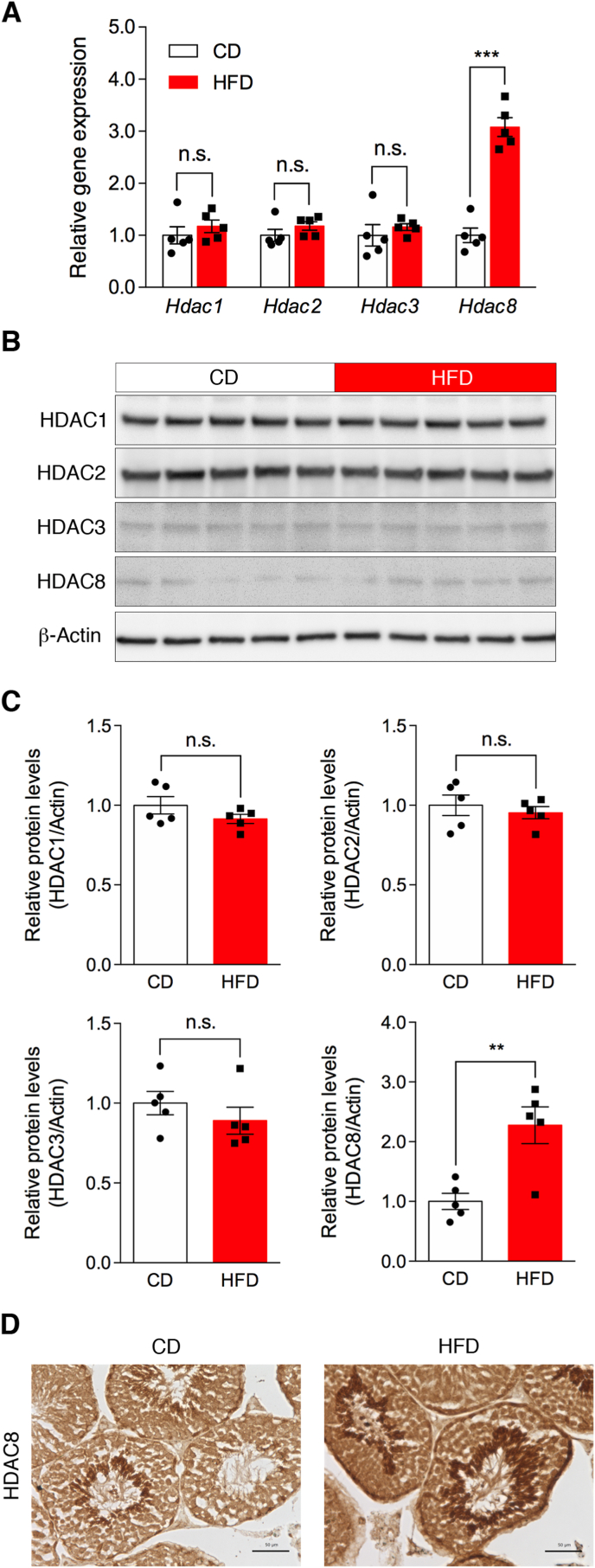
Effects of HFD on the mRNA and protein expression of the class I HDACs in the whole testis. (A) Testicular mRNA levels of the *Hdac* family in male mice fed a CD or an HFD. The amounts of mRNA, including *Hdac1*, *Hdac2*, *Hdac3*, and *Hdac8*, were normalized with reference to those of *Rn18s* mRNA. Data represent the mean ± SEM (n = 5). ***P < 0.001 (Student's *t*-test). (B) Western blot analysis of the HDAC family. Testicular tissue lysates from mice fed a CD or an HFD were subjected to western blotting with antibodies against HDAC1, HDAC2, HDAC3, or HDAC8. The equal loading of proteins was determined using an antibody against β-actin protein. (C) Quantification of HDAC family protein levels in the testis. Band densities were measured using ImageJ software and normalized to β-actin protein. (D) Immunohistochemical analysis of HDAC8 in the testes of mice fed either a CD (left) or an HFD (right). Scale bars indicate 50 μm. Data represent the mean ± SEM (n = 5). **P < 0.01 (Student's *t*-test). n.s. indicates not significant.

Steady-state levels of histone acetylation are regulated by balancing opposing histone acetyltransferase and HDAC activities. In mammals, eighteen HDACs have been identified and classified based on the homology to their yeast counterparts, and in particular, the class I enzymes (HDAC1, 2, 3, and 8) are ubiquitously expressed in most tissues, including the testes [14]. To gain molecular insight into diet-induced changes in histone acetylation levels in the testes, we investigated the effects of an HFD on the expression of class I HDACs in the whole testis. As shown in Fig. 3A, the HFD significantly increased *Hdac8* gene expression levels in the testes (CD: 100.0 ± 13.9 versus HFD: 307.9 ± 18.3, *P* < 0.001), while the *Hdac1*, *Hdac2*, and *Hdac3* mRNA levels remained unaffected. Next, we examined the protein levels of HDACs in the testes of CD- and HFD-fed mice. Western blot analysis revealed elevated immunopositive bands for HDAC8 protein in whole testis lysates obtained from HFD-fed mice compared with those from CD-fed mice (Fig. 3B). In contrast, the levels of HDAC1, 2, and 3 proteins in whole testis lysates were unaffected by HFD feeding (Fig. 3B). Densitometric measurements showed that the amount of HDAC8 protein in whole testis lysates obtained from HFD-fed mice was significantly higher than that from CD-fed mice (Fig. 3C, *P* = 0.0052). As shown in Fig. 3D, immunohistochemical analysis revealed that HDAC8 was predominantly expressed in elongated spermatids, as well as in other testicular germ cells. Increased immunopositive signals for HDAC8 were observed in the testis of mice fed an HFD. These findings suggest that decreased levels of histone H4 acetylation are associated with increased expression of HDAC8 in the testes of mice fed an HFD.

## 4 Discussion

Accumulating evidence indicates that various metabolic disorders, including obesity, cause male reproductive dysfunction. However, the molecular mechanisms underlying changes in testicular tissue homeostasis remain unclear. In this study, we demonstrated that HFD feeding in mice alters global levels of histone H4 acetylation and specifically increases HDAC8 expression at the mRNA and protein levels in the entire testis. These findings provide a basis for understanding how nutritional disturbances impair testicular tissue homeostasis.

In general, histone acetylation influences transcriptional activation by decreasing the interaction between the positively charged histone tails and the negatively charged phosphate backbone of DNA, resulting in the relaxation of the nucleosome [15,16]. It has also been shown that histone acetylation is intricately linked to the process of spermatogenesis [17,18]. In the present study, we observed that chronic HFD feeding specifically reduced the acetylation levels of histone H4 (K5, K8, K12, and K16), but not histone H3 (K9 and K14), in the whole testis lysate. These results align with previous reports showing that HFD-induced obesity reduced histone H4 acetylation levels in the entire testis of mice [10,11]. Another report showed that pan-acetylated histone H4 is detectable in testicular germ cells at the late meiotic stage [19]. Taken together, these results indicate that diet-induced obesity disrupts epigenetic signatures in the testis and leads to genomic instability and altered gene expression patterns in germ cells.

Histone acetylation levels are determined by the interplay between the enzymatic activities of histone acetyltransferases and HDACs. Mammalian HDACs are generally classified into three classes (I–III) based on their sequence homology with their yeast counterparts [14]. Class I HDACs (HDAC1, 2, 3, and 8) are expressed in most tissues, and differential expression patterns of class I HDACs have been reported in the testicular tissue. HDAC1 proteins are primarily found in spermatocytes and persist in the spermatid stage [19]. HDAC2 expression is strongly observed in primary spermatocytes and weakens in subsequent stages of spermatogenesis [19]. HDAC3 is readily detected in spermatogonia and somatic Sertoli cells, but not in leptotene, zygotene, or early pachytene spermatocytes [20]. Although little information is available regarding HDAC8 expression in testicular germ cells, a recent report showed that the *Hdac8* gene is highly expressed in c-kit-negative undifferentiated spermatogonia, downregulated in c-kit-positive differentiated spermatogonia, and continuously expressed in pre-leptotene/zygotene spermatocytes [21]. The present study is the first to show that HDAC8 is significantly upregulated at the mRNA and protein levels in the entire testis after chronic HFD exposure. We also found increased immunopositive signals of HDAC8 in the testis of HFD-fed mice. However, the regulatory mechanisms of expression remain unknown. These results suggest that HFD feeding alters the expression of HDAC8 in testicular germ cells, leading to abnormal histone acetylation during spermatogenesis. Further studies are needed to determine whether inhibiting HDAC8 activity reverses the alteration of histone acetylation levels in the testis due to HFD exposure.

While most histone proteins are replaced by protamines during sperm maturation, approximately 5% of histone proteins in mouse sperm and 10% in human sperm are retained [22]. These retained histone proteins are believed to play a role in spermatogenesis, embryo development, and the transmission of information across generations. We recently reported that exposing male mice to an HFD affects triglyceride metabolism, accompanied by changes in the expression of genes related to lipid metabolism in the liver and white adipose tissue of their offspring [12]. The mechanisms by which paternal nutrition and metabolic status affect progeny phenotypes are largely unknown, but epigenetic regulation, namely the posttranslational modification of histone proteins in sperm, has been shown to contribute to paternal intergenerational inheritance [23,24]. Notably, it remains to be determined whether inhibiting HDAC8 activity also improves the intergenerational transmission of paternal metabolic disorders to the offspring.

A previous report showed that HFD exposure in mice induced a time-dependent decrease in testicular weight and testicular histological abnormalities, including disrupted seminiferous tubules, increased tubular vacuolization, and enlarged interstitial spaces, leading to Leydig cell dystrophy [25]. However, no differences in testicular weight or morphology were observed in our models (Fig. 1D and E). These discrepancies may be attributed to differences in dietary fat composition, feeding duration, or mouse strain. Future studies should consider how different environmental stimuli and genetic backgrounds affect testicular tissue homeostasis and epigenetic signatures.

In conclusion, our findings demonstrate that an HFD affects epigenetic marks, such as the posttranslational modifications of histone proteins, and alters the expression of regulatory enzymes in the testes. These results suggest that nutritional and metabolic disturbances cause epigenetic deregulation in the testes, resulting in male reproductive dysfunction and potentially impacting the metabolic health of offspring.

## Data statement

All data supporting the results of this study will be made available by the corresponding author upon reasonable request.

## CRediT authorship contribution statement

**Shu Aizawa:** Conceptualization, Funding acquisition, Investigation, Project administration, Resources, Supervision, Writing – original draft, Writing – review & editing. **Hikari Ohno:** Data curation, Formal analysis, Investigation, Visualization. **Yutaka Yamamuro:** Funding acquisition, Methodology, Resources.

## Declaration of competing interest

The authors declare no competing interests.

## Data Availability

Data will be made available on request.
