# Supplementary material for: Chronic high-fat diet decreases global histone H4 acetylation and increases HDAC8 expression in mouse testes
Source: Biochem Biophys Rep. 2026 May 20;46:102642. doi: 10.1016/j.bbrep.2026.102642 (PMC13214310; doi:10.1016/j.bbrep.2026.102642)
Supplement: Multimedia component 1 [file mmc1.docx]

**Supplementary Table S1.** The antibodies used for western blot analysis

| ANTIBODIES | SOURCE | IDENTIFIER |
| --- | --- | --- |
| Acetyl-Histone H3 (K4 and K9)  (1:1,000) | Merck Millipore | Catalog #: 06-599 |
| Acetyl-Histone H4 (K5, K8, K12 and K16)  (1:1,000) | Merck Millipore | Catalog #: 06-866 |
| HDAC1  (1:1,000) | BioVision, Inc. | Catalog #: 3601 |
| HDAC2  (1:1,000) | BioVision, Inc. | Catalog #: 3602 |
| HDAC3  (1:1,000) | BioVision, Inc. | Catalog #: 3603 |
| HDAC8  (1:1,000) | Proteintech Group Inc. | Catalog #: 17548-1-AP |
| β-actin  (1:5,000) | Sigma-Aldrich | Catalog #: A1978 |
| Anti-rabbit IgG HRP-linked antibody  (1:10,000) | Cell Signaling Technology | Catalog #: 7074 |
| Anti-mouse IgG, HRP-linked antibody  (1:10,000) | Cell Signaling Technology | Catalog #: 7076 |
