# Supplementary material for: Chronic high-fat diet decreases global histone H4 acetylation and increases HDAC8 expression in mouse testes
Source: Biochem Biophys Rep. 2026 May 20;46:102642. doi: 10.1016/j.bbrep.2026.102642 (PMC13214310; doi:10.1016/j.bbrep.2026.102642)
Supplement: Multimedia component 2 [file mmc2.docx]

**Supplementary Table S2.** The primer sequences used for real-time quantitative PCR analysis

| Symbol | Forward/Reverse | Primer sequences |
| --- | --- | --- |
| *Hdac1*  *Hdac2*  *Hdac3*  *Hdac8*  *Rn18s* | Forward  Reverse  Forward  Reverse  Forward  Reverse  Forward  Reverse  Forward  Reverse | 5’-CCCTAATGAGCTGCCCTACA-3’  5’-TTCTGGTTGGTCATGTTGGA-3’  5’-CCATTCGAGCATCAGACAAA-3’  5’-CGACCTCCTTCACCTTCATC-3’  5’-GATGCTGTGTCCCCAAGAGT-3’  5’-TTCCAAACCCTTCACCAGAG-3’  5’-GGGAATCTGAAGCATGTGGT-3’  5’-TTTCCCCTGCAGTCACAAAT-3’  5’-CGCCGCTAGAGGTGAAATTCT-3’  5’-CGAACCTCCGACTTTCGTTCT-3’ |
