# Supplementary material for: Chronic high-fat diet decreases global histone H4 acetylation and increases HDAC8 expression in mouse testes
Source: Biochem Biophys Rep. 2026 May 20;46:102642. doi: 10.1016/j.bbrep.2026.102642 (PMC13214310; doi:10.1016/j.bbrep.2026.102642)
Supplement: Multimedia component 3 [file mmc3.docx]

**
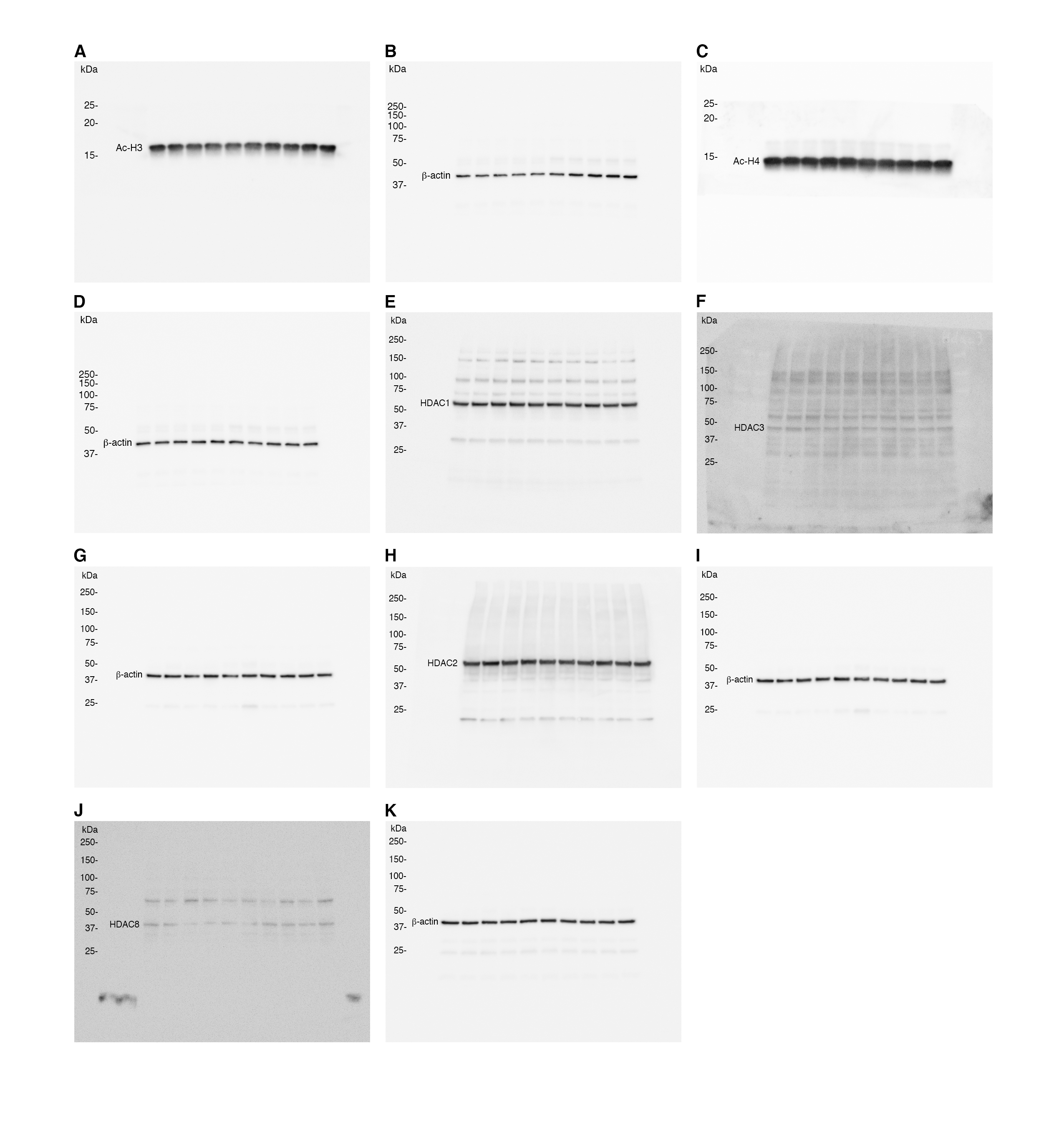
**

**Supplementary Figure S1. Full-length images of the western blot analysis.** Antibodies against (A) Ac-H3, (B) β-actin for Ac-H3 normalization, (C) Ac-H4, (D) β-actin for Ac-H4 normalization, (E) HDAC1, (F) HDAC3, (G) β-actin for HDAC1 and HDAC3 normalization, (H) HDAC2, (I) β-actin for HDAC2 normalization, (J) HDAC8, and (K) β-actin for HDAC8 normalization.
